# Diet in irritable bowel syndrome

**DOI:** 10.1186/s12937-015-0022-3

**Published:** 2015-04-14

**Authors:** Magdy El-Salhy, Doris Gundersen

**Affiliations:** 1 Department of Medicine, Section for Gastroenterology, Stord Hospital, Stord, Norway; 2 Department of Clinical Medicine, Section for Gastroenterology, University of Bergen, Box 4000, 54 09 Stord, Norway; 3 Department of Medicine, National Centre for Functional Gastrointestinal Disorders, Haukeland University Hospital, Bergen, Norway; 4 Department of Research, Helse-Fonna, Haugesund Hospital, Haugesund, Norway

**Keywords:** Appetite, Non-celiac gluten sensitivity, Endocrine cells

## Abstract

Irritable bowel syndrome (IBS) is a common chronic gastrointestinal disorder that is characterized by intermittent abdominal pain/discomfort, altered bowel habits and abdominal bloating/distension. This review aimed at presenting the recent developments concerning the role of diet in the pathophysiology and management of IBS. There is no convincing evidence that IBS patients suffer from food allergy/intolerance, and there is no evidence that gluten causes the debated new diagnosis of non-coeliac gluten sensitivity (NCGS). The component in wheat that triggers symptoms in NCGS appears to be the carbohydrates. Patients with NCGS appear to be IBS patients who are self-diagnosed and self-treated with a gluten-free diet. IBS symptoms are triggered by the consumption of the poorly absorbed fermentable oligo-, di-, monosaccharides and polyols (FODMAPs) and insoluble fibre. On reaching the distal small intestine and colon, FODMAPS and insoluble fibre increase the osmotic pressure in the large-intestine lumen and provide a substrate for bacterial fermentation, with consequent gas production, abdominal distension and abdominal pain or discomfort. Poor FODMAPS and insoluble fibres diet reduces the symptom and improve the quality of life in IBS patients. Moreover, it changes favourably the intestinal microbiota and restores the abnormalities in the gastrointestinal endocrine cells. Five gastrointestinal endocrine cell types that produce hormones regulating appetite and food intake are abnormal in IBS patients. Based on these hormonal abnormalities, one would expect that IBS patients to have increased food intake and body weight gain. However, the link between obesity and IBS is not fully studied. Individual dietary guidance for intake of poor FODMAPs and insoluble fibres diet in combination with probiotics intake and regular exercise is to be recommended for IBS patients.

## Introduction

Irritable bowel syndrome (IBS) is a common chronic gastrointestinal disorder that affects 5–20% of the general population [1-15]. IBS is usually diagnosed at a young age (i.e. <50 years of age) and is more common in females than males [1,3,5,6,16]. This condition reduces considerably the patients’ quality of life, although it is not known to progress to a more serious disease or to cause death [1,17-19].

IBS patients suffer from intermittent abdominal pain/discomfort, altered bowel habits and abdominal bloating/distension [1,2]. Patients believe that their symptoms are triggered by certain food items such as milk and milk products, wheat products, caffeine, cabbage, onion, peas, beans, hot spices, and fried and smoked food [20-23]. Some IBS patients avoiding several foodstuffs, but there does not appear to be any difference between them and the general population regarding the intake of energy, carbohydrates, proteins and fats [23-29]. However, one study found that 62% of IBS patients had either limited or excluded certain food items from their daily diet, and of these 12% were at risk of long-term nutritional deficiencies [30].

The role of diet in the development of IBS symptoms and dietary management as a tool for controlling these symptoms has been the subject of several reviews [20,29,31-36]. The aim of this review was to present the recent developments concerning the role of diet in the pathophysiology and management of IBS.

### Diet and the pathophysiology of IBS

It is generally accepted that diet plays an important role in the pathophysiology of IBS [27,36-45]. Several factors have been proposed for explaining how diet influences IBS, such as food allergy/intolerance, poorly absorbed carbohydrates and fibre, and the comorbidity of obesity and IBS.

#### Food allergy/intolerance

Food allergy occurs in 6–8% of children and 1–4% of adults [46]. The food allergy reaction, which is mediated by immunoglobulin E, occurs within 2 hours of ingesting the offending food item, and manifests as swelling, itching, hives, wheezing, nausea, vomiting, diarrhoea, abdominal pain and collapse. There is no evidence that such an allergic reaction takes place in IBS [47-54]. A large proportion of IBS patients complain of subjective intolerance to various foods [20,21,52,55-60]. Food intolerance is a non-toxic, non-immune-mediated reaction to bioactive chemicals in food such as histamines, sulphites and monosodium glutamate, with symptoms usually manifesting outside the gastrointestinal tract. There is no documented proof that such intolerance occurs in IBS [42,54].

Non-coeliac gluten sensitivity (NCGS) has recently received attention from the mass media and the general public, and has become confused with the popular speculation that the high carbohydrate content of wheat is responsible for negative health aspects such as obesity [61]. NCGS is defined as having gastrointestinal and extra-gastrointestinal IBS-like symptoms without coeliac disease or wheat allergy, but with the symptoms being relieved by a gluten-free diet (GFD) and relapsing on gluten challenge [62-69]. The prevalence of NCGS has been reported as 0.55–6% of the USA population [64,70].

For more than 3 decades, patients with abdominal pain, diarrhoea and small-intestine biopsy findings with no significant changes have experienced symptom relief on a GFD with return of the symptoms after a gluten challenge [71,72]. Similar results have been reported in patients with non-celiac IBS-like symptoms [73-75]. This was confirmed by double-blind randomized placebo-controlled studies [76,77]. There is disagreement as to whether or not NCGS patients have low-grade inflammation and abnormal intestinal permeability [74,77-82].

It is noteworthy that in studies showing beneficial effects on symptoms in NCGS [74,76,77], those effects were actually the result of wheat withdrawal rather than withdrawal of gluten [83]. In a placebo-controlled, crossover study of patients with IBS-like symptoms on a self-imposed GFD [84], the gastrointestinal symptoms consistently and significantly improved when consuming a diet with reduced fermentable oligo-, di-, monosaccharides and polyols (FODMAPs), and these symptoms were not worsened by either a low- or high-dose challenge with gluten. It therefore seems that the carbohydrate content (fructans and galactans) of wheat rather than gluten is responsible for triggering NCGS symptoms. Furthermore, in those who believed that they had NCGS, 24% had uncontrolled symptoms despite consuming a GFD, 27% did not follow a GFD alone, and 65% avoided other foods that contain high levels of FODMAPs [85]. These findings lend further support to the idea that NCGS symptoms are not triggered by the gluten in wheat.

The basic description of NCGS [67] is the same as that of IBS. Both NCGS and IBS patients have the same gastrointestinal and extra-gastrointestinal symptoms that are triggered by wheat consumption. NCGS patients have been reported to have high frequency of immunoglobulin G (IgG)/immunoglobulin A (IgA) antigliadin antibodies (AGA) and a stronger association with human leucocyte antigen-DQ2 (DQ2) and -DQ8 (DQ8) [76]. The prevalence of positivity for IgG/IgA AGA, with negative tissue transglutaminase or deamidated gliadin peptide antibodies, in the blood of IBS patients has been reported to be 5–17% [86-88] or as high as about 50% [65,89]. Serum AGA has been reported to have a good sensitivity but a low specificity for coeliac disease [90], and 12–15% of serum samples from healthy subjects are positive for AGA [86,87,90,91]. Moreover, DQ2 and DQ8 are common in the general population. It appears that NCGS patients are IBS patients with a self-diagnosis and who self-treat with a GFD. It is noteworthy in this context that 20–70% of IBS patients complain of subjective intolerance to various foods [20,21,52,55-60].

#### Poorly absorbed carbohydrates and fibre

The triggering of symptoms in IBS patients by certain foodstuff has been attributed to indigestible and poorly absorbed short-chain carbohydrates, FODMAPs [42,92-94] and FODMAPs intake is hypothesized to be one factor among others for IBS aetiology. These short-chain sugars include fructose, lactose, sugar alcohols (sorbitol, maltitol, mannitol, xylitol and isomalt), fructans and galactans [42]. FODMAPs occur in a wide range of foods, including wheat, rye, vegetables, fruits and legumes [95-97]. A significant proportion of these carbohydrates enter the distal small intestine and colon, where they exert osmotic effects in the large-intestine lumen, increasing its water content and providing a substrate for bacterial fermentation, with consequent gas production [92,95,98]. The produced gas causes abdominal distension and abdominal pain/discomfort. FODMAPs have been found to trigger gastrointestinal symptoms in IBS, and a low-FODMAPs diet reduces the symptoms and improves the patient’s quality of life [23,29,93,94,99-102]. Recent studies have shown that the mechanisms by which FODMAPs exert their effects are more complicated than originally thought. A low-FODMAPs diet appears to induce favourable changes in the intestinal microbiota [103] and gastrointestinal endocrine cells [104-107].

It has been reported that changing from typical Australian food to a low-FODMAPs diet changed the intestinal microbiota [103]. Thus, a low-FODMAPs diet in healthy subjects and IBS patients reduced the total bacterial abundance, while a typical Australian diet increased the relative abundance of butyrate-producing *Clostridiun cluster XIVa* and the mucus-associated *Akkermansia muciniphia*, and reduced *Ruminococcus torques* [103].

Several types of endocrine cell in all segments of the gastrointestinal tract of IBS patients are abnormal [108-129]. The gastrointestinal endocrine cells interact and integrate with each other, with the enteric nervous system and with the afferent and efferent nerve fibres of the central nervous system, in particular the autonomic nervous system [42,130-132]. These cells regulate several functions of the gastrointestinal tract, including sensation, motility, secretion, absorption, local immune defence and food intake (by affecting appetite) [42,131-134]. The abnormalities in the gastrointestinal endocrine cells are considered to play a major role in the development of symptoms in IBS, and therefore represent future targets for treatment [43,135]. Switching from a typical Norwegian diet to a low-FODMAPs diet was shown to lead to normalization of the endocrine cells in the stomach and large intestines [104-107].

A low intake of dietary fibre was initially believed to be the cause of IBS [136]. In clinical settings the increase in dietary fibre intake in IBS patients has been found to increase abdominal pain, bloating and abdominal distension. A meta-analysis of 12 trials revealed that IBS patients treated with increased fibre intake had no improvement in symptoms compared to placebo or a low-fibre diet [137]. However, it has been reported that water-soluble fibre—but not insoluble fibre—improves the symptoms [138,139].

#### Obesity and IBS

As mentioned above, IBS patients tend to avoid certain food items that they associate with the onset of their symptoms. There has been some concern that the onset of IBS symptoms upon ingesting certain foods would reduce the amount of food consumed and thereby lead to malnutrition [30]. However, whereas an association between low BMI and IBS in 367 patients with IBS has been reported [140], in another report most of the 330 IBS patients examined were either normal or overweight [20]. In a recent comprehensive review, the association between IBS and obesity was found to be controversial, and the author concluded that obesity and IBS might be linked [141].

Appetite is regulated by a large number of hormones, several of which are secreted by gastrointestinal endocrine cells [142]. The gastrointestinal hormones exert their effects by acting upon the appetite control centre in the hypothalamus [142]. The arcuate nucleus (ARC) lies in the median eminence, which lacks a complete blood barrier, making the ARC particularly susceptible to hormones circulating in the blood [142-145]. The ARC is the centre that integrates the neurological and blood-borne signals [142-145]. The brain reward system in the midbrain controls hedonic feeding (i.e. the consumption of palatable food), which is modulated by blood-borne signals [145].

**Figure 1:**
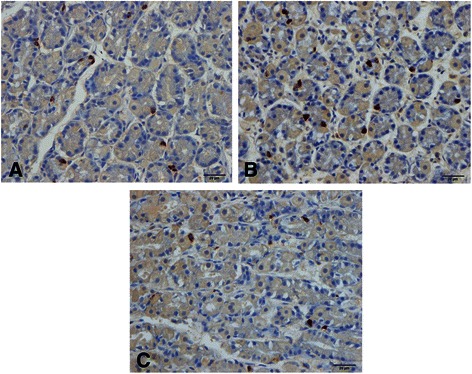
Ghrelin-immunoreactive cells in the oxyntic mucosa of a healthy subject **(A)**, in a patient with IBS-D **(B)** and in a patient with IBS-C **(C)**.

**Figure 2:**
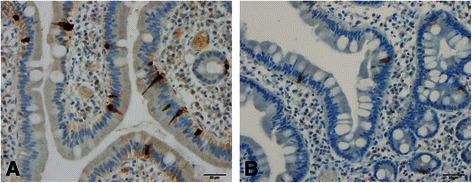
Duodenal CCK cells in a healthy subject **(A)** and in a patient with IBS **(B)**.

**Figure 3:**
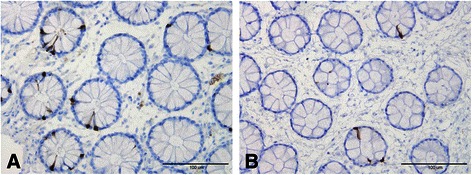
PYY cells in the colon of a healthy subject **(A)** and in a patient with IBS **(B)**.

**Figure 4:**
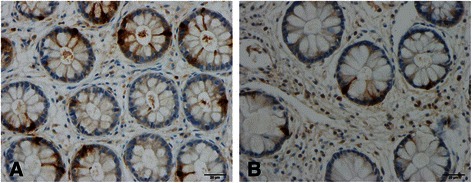
Rectal enteroglucagon cells in a healthy subject **(A)** and in a patient with IBS **(B)**.

**Figure 5:**
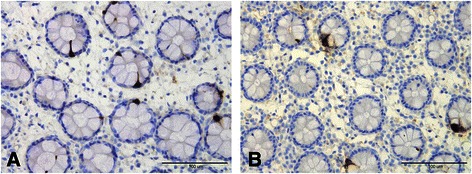
Serotonin cells in the colon of a healthy subject **(A)** and in a patient with IBS **(B)**.

The following five gastrointestinal endocrine cell types that secrete hormones that regulate appetite are abnormal in patients with IBS: ghrelin, cholecystokinin (CCK), peptide YY (PYY), enteroglucagon (oxyntomodulin) and serotonin (Table 1 and Figures 1, 2, 3, 4 and 5) [108-110,112-115,132,146-149]. The endocrine cells in the oxyntic mucosa are the main source of circulating ghrelin, although small amounts do occur in the small and large intestines as well as in the ARC of the hypothalamus [144,150-153]. Ghrelin has several roles, such as regulating the release of pituitary growth hormone and accelerating gastric and intestinal motility [150-166]. Moreover, ghrelin increases appetite and feeding; central or peripheral administration of ghrelin stimulates the consumption of food and body weight gain [150]. CCK stimulates gallbladder contraction, intestinal motility and pancreatic exocrine secretion, and inhibits gastric motility and food consumption [131]. The anorexigenic action of CCK occurs via the CCK-B (CCK-2) receptor, which is the predominant receptor type in the brain [167-183]. PYY release is proportional to the composition (including calorie content) of a particular meal, and its infusion reduces the consumption of food [184,185]. PYY binds to Y2 receptors localized on the presynaptic terminals of neuropeptide Y and to agouti-related protein neurons in the hypothalamus, which causes inactivation of these neurons, resulting in anorexia [186]. Moreover, PYY is the main regulator of the ileal brake and consequently inhibits the consumption of further food once nutrients reach the terminal ileum [187-194]. Enteroglucagon reduces gastric motility and secretion [195-200] and, similar to PYY, the amount released into the bloodstream is proportional to the calories ingested [201,202]; however, enteroglucagon seems to have only a modest anorexigenic effect [145]. Serotonin has also been reported to have an anorexigenic effect [203].

**Table 1:** **Abnormalities in the gastrointestinal endocrine cells that regulate appetite in IBS patients**

Gastrointestinal segment	Hormone	Cell density			Hormone function
		IBS-D	IBS-M	IBS-C	
Stomach	Ghrelin	Increased	Unchanged	Decreased	Orexigenic (increases appetite)
	Serotonin	Increased	Unchanged	Decreased	Anorexigenic (decreases appetite)
Duodenum	CCK	Decreased	Unchanged	Unchanged	Anorexigenic (decreases appetite)
	Serotonin	Unchanged	Unchanged	Unchanged	See above
Ileum	PYY	Unchanged	Unchanged	Increased	Anorexigenic (decreases appetite)
	Serotonin	Decreased	Decreased	Decreased	See above
Colon	PYY	Decreased	Unknown	Decreased	See above
	Serotonin	Decreased	Unknown	Decreased	See above
Rectum	PYY	Decreased	Decreased	Decreased	See above
	Enteroglucagon	Decreased	Unknown	Decreased	Anorexigenic (decreases appetite)
	Serotonin	Unchanged	Unknown	Unchanged	See above

IBS-D, IBS patients with diarrhoea as the predominant symptom; IBS-M, IBS patients with alternating diarrhea and constipation; IBS-C, IBS patients with constipation as the predominant symptom.

Whereas the ghrelin cell density is increased in IBS patients with diarrhoea as the predominant symptom (IBS-D), it is reduced in IBS patients with constipation as the predominant symptom (IBS-C). It is therefore reasonable to assume that IBS-D patients would have a greater appetite and food intake than those with IBS-C. In both IBS subtypes, the densities of the four endocrine cell types that produce anorexigenic hormones—namely CCK, PYY, enteroglucagon and serotonin—are reduced. Thus, the changes in the gastrointestinal endocrine cells regulating appetite in IBS patients favour an increase in food consumption. BMI and appetite in IBS patients have not been studied in detail, and the available data are controversial. It is not clear whether IBS patients have a greater appetite, which is opposed by the avoidance of eating because of worsening of symptoms upon eating. Further studies are needed to clarify this issue.

### Diet and management of IBS

As mentioned above, it is recommended that IBS patients consume a diet that is poor in FODMAPs and insoluble fibre. IBS patients make a conscious choice to avoid certain food items, some of which are rich in FODMAPs, but they may also consume large amounts of other food items that are rich in FODMAPs and avoid food sources that are important to health maintenance [23]. Dietary guidance is therefore important for IBS patients [23,99]. Furthermore, in our experience individual dietary guidance is preferable due to the wide variety of individual tolerances to different FODMAPs-rich food items, probably due to the intestinal microbiota differing between individuals.

Consuming probiotics increases the tolerance for FODMAPs-rich foodstuffs, and adding regular exercise amplifies the beneficial effects of such a diet [1,204]. People in numerous countries (including Norway) rely on bread and wheat products for a substantial part of their diet [205]. Gluten-free bread (mostly made of rice/corn) contains 0.19 g/100 g fructans, and bread made with spelt flour contains 0.14 g/100 g [96]. Either gluten-free products or spelt products can be consumed. Many of our patients consume spelt bread and spelt products rather than gluten-free products, with satisfactory results. The protein content of spelt flour is also 16% lower in terms of gluten than that in wheat [206].

## Conclusion

**Figure 6:**
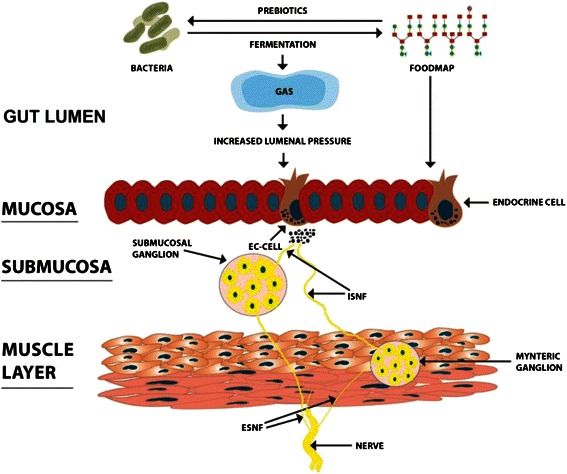
Schematic illustration for the possible mechanisms by which FODMAPs can trigger IBS symptoms. Upon reaching the large intestine FODMAPs can exert direct or indirect effect on the intestinal endocrine cells. They act as prebiotics and change the intestinal flora and they are fermented by the intestinal microbiota with gas production. The production of gas increases the luminal pressure and stimulates the release of serotonin from serotonin (EC) cells. Serotonin act on the intrinsic sensory nerve fibres (ISNF) of the submucosal and myenteric ganglia, which in turn convey the activation to the extrinsic sensory nerve fibres (ESNF) to the central nervous system.

Diet plays a major role in the pathophysiology of IBS and is a powerful tool for managing IBS. There is no convincing evidence that IBS patients suffer from food allergy/intolerance, and there is evidence that NCGS is caused by the fructans in wheat, rather than by gluten. NCGS patients appear to be IBS patients who have self-diagnosed and self-treated with a GFD. Food items that are rich in poorly absorbed short-chain carbohydrates (FODMAPs) and insoluble fibre trigger IBS symptoms. There appear to be several mechanisms by which these food items exert this effect (Figure 6):

1. Upon entering the distal small intestine and colon, they increase the osmotic pressure and provide a substrate for bacterial fermentation, resulting in gas production, abdominal distension and abdominal pain/discomfort.
2. They are prebiotics that favour the colonization of the large intestine with *Clostridium* bacteria, which produce gas on fermentation.
3. They affect the gastrointestinal endocrine cells that regulate gastrointestinal sensation, motility, secretion and absorption, as well as local immune defence and food consumption.

The link between obesity and IBS is an interesting area that needs to be explored further. This is of particular interest since IBS patients have an increased density of ghrelin cells, which increases appetite, stimulates the consumption of food and body weight gain, and have decreased densities of the four endocrine cells that produce anorexigenic hormones, namely CCK, PYY, enteroglucagon and serotonin.

A diet that is poor in FODMAPs and insoluble fibre reduces the symptoms and improves the quality of life of IBS patients. Individual dietary guidance is necessary to identify a suitable diet to which the patient is likely to adhere to in the long term. Combining this diet with probiotics and regular exercise will amplify the effect of such a diet.

## Abbreviations

AGA: Anti-gliadin antibodies
ARC: Arcuate nucleus
FODMAPs: Fermentable oligo-, di-, monosaccharides and polyols
GFD: Gluten-free diet
IBS: Irritable bowel syndrome
IBS-D: IBS patients with diarrhoea as the predominant symptom
IBS-C: IBS patients with constipation as the predominant symptom
NCGS: Non-coeliac gluten sensitivity
